# *N*-(2-Chloro­pyrimidin-4-yl)-2-methyl-2*H*-indazol-6-amine methanol monosolvate

**DOI:** 10.1107/S1600536811017831

**Published:** 2011-05-14

**Authors:** Xiang-Chuan Pang, Xin-Hua Deng, Yuan Sun

**Affiliations:** a College of Materials Science and Engineering, Tianjin Polytechnic University, Tianjin 300160, People’s Republic of China

## Abstract

In the title compound, C_12_H_10_ClN_5_·CH_3_OH, the indazole ring system and the pyrimidine ring make a dihedral angle of 23.86 (4)°. In the crystal, the components are linked by N—H⋯O and O—H⋯N hydrogen bonds into chains propagated in [010]. Inter­molecular π–π inter­actions [centroid–centroid distances = 3.6404 (9), 3.6725 (9) and 3.4566 (9) Å] between the rings of neighbouring chains also stabilize the crystal packing.

## Related literature

The title compound was obtained in a continuation of our studies of derivatives of the anti­tumor agent pazopanib (systematic name 5-[[4-[(2,3-dimethyl-2*H*-indazol-6-yl)methyl­amino]-2-pyrimidin­yl]amino]-2-methyl­benzolsulfonamide), during which we determined the crystal structure of the related compound *N*-(2-chloro­pyrimidin-4-yl)-*N*,2-dimethyl-2*H*-indazol-6-amine, see: Qi *et al.* (2010 ▶).
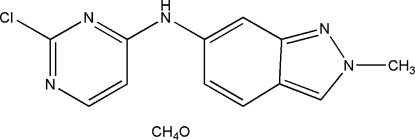


## Experimental

### 

#### Crystal data

C_12_H_10_ClN_5_·CH_4_O
*M* *_r_* = 291.74
Monoclinic, 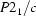

*a* = 6.9327 (8) Å
*b* = 17.613 (2) Å
*c* = 11.4883 (16) Å
β = 106.690 (8)°
*V* = 1343.7 (3) Å^3^
*Z* = 4
Mo *K*α radiation
μ = 0.29 mm^−1^
*T* = 113 K
0.34 × 0.28 × 0.12 mm

#### Data collection

Rigaku Saturn CCD area-detector diffractometer
Absorption correction: multi-scan (*CrystalClear*; Rigaku/MSC, 2005 ▶) *T*_min_ = 0.909, *T*_max_ = 0.966
13795 measured reflections
3193 independent reflections
2982 reflections with *I* > 2σ(*I*)
*R*_int_ = 0.057

#### Refinement

*R*[*F* ^2^ > 2σ(*F* ^2^)] = 0.039
*wR*(*F* ^2^) = 0.102
*S* = 1.11
3193 reflections
191 parameters
H atoms treated by a mixture of independent and constrained refinement
Δρ_max_ = 0.46 e Å^−3^
Δρ_min_ = −0.22 e Å^−3^

### 

Data collection: *CrystalClear* (Rigaku/MSC, 2005 ▶); cell refinement: *CrystalClear*; data reduction: *CrystalClear*; program(s) used to solve structure: *SHELXS97* (Sheldrick, 2008 ▶); program(s) used to refine structure: *SHELXL97* (Sheldrick, 2008 ▶); molecular graphics: *SHELXTL* (Sheldrick, 2008 ▶); software used to prepare material for publication: *SHELXTL*.

## Supplementary Material

Crystal structure: contains datablocks I, global. DOI: 10.1107/S1600536811017831/cv5083sup1.cif

Structure factors: contains datablocks I. DOI: 10.1107/S1600536811017831/cv5083Isup3.hkl

Supplementary material file. DOI: 10.1107/S1600536811017831/cv5083Isup3.cml

Additional supplementary materials:  crystallographic information; 3D view; checkCIF report

## Figures and Tables

**Table 1:** Hydrogen-bond geometry (Å, °)

*D*—H⋯*A*	*D*—H	H⋯*A*	*D*⋯*A*	*D*—H⋯*A*
O1—H1⋯N4^i^	0.80 (2)	2.04 (2)	2.8394 (15)	176 (2)
N3—H3*A*⋯O1	0.846 (19)	2.110 (19)	2.9452 (14)	168.8 (16)

Symmetry code: (i) 
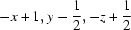
.

**Fig. 1.**
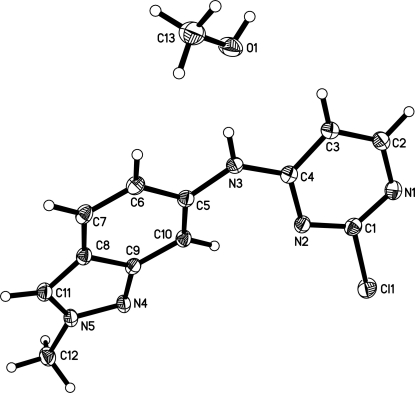
The molecular structure of (I). Displacement ellipsoids are drawn at the 50% probability level.

## supplementary crystallographic information

### Comment

In continuation of our studies of derivatives of antitumor agent pazopanib (Qi *et al.*, 2010), we obtained the title compound (I).

In (I) (Fig. 1), all bond lengths and angles are normal and correspond to those reported by Qi *et al.* (2010). In *N*-(2-chloropyrimidin-4-yl)-2-methyl-2*H*-indazol-6-amine (*M*) molecule, the indazole and pyrimidin fragments form a dihedral angle of 23.86 (4)°.

In the crystal structure, *M* and methanol molecules are linked by N—H···O and O—H···N hydrogen bonds (Table 2) into chains propagated in [010]. Intermolecular π—π interactions (Table 1) between the rings from the neighbouring chains stabilize the crystal packing.

### Experimental

To a stirred solution of the 2-methyl-2*H*-indazol-6-amine (10 g, 0.07 mol) and NaHCO_3_ (12 g, 0.14 mol) in ethanol (250 ml) was added 2,4-dichloropyrimidine (12 g, 0.08 mol) at room temperature. After the reaction was heated for four hours, the suspension was cooled to room temperature, filtered and washed thoroughly with ethyl acetate. The filtrate was concentrated under reduced pressure to get off-white solid as crude product. The solid was dissolved in methanol 40 ml at 293 k, then colourless crystals were generated slowly.

### Refinement

C-bound H atoms were geometrically positioned (C—H 0.95–0.98 Å), and refined as riding with *U*_iso_ = 1.2–1.5*U*_eq_(C). The H atoms of N—H and O—H were found from difference map and isotropically refined.

### Crystal data

**Table:**

C_12_H_10_ClN_5_·CH_4_O	*F*(000) = 608
*M**_r_* = 291.74	*D*_x_ = 1.442 Mg m^−^^3^
Monoclinic, *P*2_1_/*c*	Mo *K*α radiation, λ = 0.71073 Å
Hall symbol: -P 2ybc	Cell parameters from 4513 reflections
*a* = 6.9327 (8) Å	θ = 1.9–27.9°
*b* = 17.613 (2) Å	µ = 0.29 mm^−^^1^
*c* = 11.4883 (16) Å	*T* = 113 K
β = 106.690 (8)°	Prism, colorless
*V* = 1343.7 (3) Å^3^	0.34 × 0.28 × 0.12 mm
*Z* = 4	

### Data collection

**Table:**

Rigaku Saturn CCD area-detector diffractometer	3193 independent reflections
Radiation source: rotating anode	2982 reflections with *I* > 2σ(*I*)
multilayer	*R*_int_ = 0.057
Detector resolution: 14.63 pixels mm^-1^	θ_max_ = 27.9°, θ_min_ = 2.2°
ω and φ scans	*h* = −9→9
Absorption correction: multi-scan (*CrystalClear*; Rigaku/MSC, 2005)	*k* = −23→23
*T*_min_ = 0.909, *T*_max_ = 0.966	*l* = −15→14
13795 measured reflections	

### Refinement

**Table:**

Refinement on *F*^2^	Primary atom site location: structure-invariant direct methods
Least-squares matrix: full	Secondary atom site location: difference Fourier map
*R*[*F*^2^ > 2σ(*F*^2^)] = 0.039	Hydrogen site location: inferred from neighbouring sites
*wR*(*F*^2^) = 0.102	H atoms treated by a mixture of independent and constrained refinement
*S* = 1.11	*w* = 1/[σ^2^(*F*_o_^2^) + (0.0548*P*)^2^ + 0.3525*P*] where *P* = (*F*_o_^2^ + 2*F*_c_^2^)/3
3193 reflections	(Δ/σ)_max_ < 0.001
191 parameters	Δρ_max_ = 0.46 e Å^−^^3^
0 restraints	Δρ_min_ = −0.22 e Å^−^^3^

### Special details

**Table:**

Geometry. All e.s.d.'s (except the e.s.d. in the dihedral angle between two l.s. planes) are estimated using the full covariance matrix. The cell e.s.d.'s are taken into account individually in the estimation of e.s.d.'s in distances, angles and torsion angles; correlations between e.s.d.'s in cell parameters are only used when they are defined by crystal symmetry. An approximate (isotropic) treatment of cell e.s.d.'s is used for estimating e.s.d.'s involving l.s. planes.
Refinement. Refinement of *F*^2^ against ALL reflections. The weighted *R*-factor *wR* and goodness of fit *S* are based on *F*^2^, conventional *R*-factors *R* are based on *F*, with *F* set to zero for negative *F*^2^. The threshold expression of *F*^2^ > σ(*F*^2^) is used only for calculating *R*-factors(gt) *etc*. and is not relevant to the choice of reflections for refinement. *R*-factors based on *F*^2^ are statistically about twice as large as those based on *F*, and *R*- factors based on ALL data will be even larger.

### Fractional atomic coordinates and isotropic or equivalent isotropic displacement parameters (Å^2^)

**Table:**

	*x*	*y*	*z*	*U*_iso_*/*U*_eq_	
Cl1	−0.00948 (5)	0.910378 (17)	−0.00877 (3)	0.02682 (12)	
N1	−0.00275 (16)	0.77456 (6)	−0.09446 (10)	0.0196 (2)	
N2	0.16759 (15)	0.79520 (6)	0.11655 (9)	0.0171 (2)	
N3	0.34415 (16)	0.69626 (6)	0.23692 (9)	0.0169 (2)	
N4	0.65827 (16)	0.92220 (6)	0.46761 (9)	0.0182 (2)	
N5	0.75805 (16)	0.92466 (6)	0.58823 (9)	0.0182 (2)	
C1	0.05934 (19)	0.81451 (7)	0.00587 (11)	0.0183 (2)	
C2	0.05172 (19)	0.70044 (7)	−0.07866 (11)	0.0196 (3)	
H2	0.0110	0.6673	−0.1466	0.023*	
C3	0.16207 (18)	0.67115 (7)	0.02996 (11)	0.0181 (2)	
H3	0.1953	0.6187	0.0388	0.022*	
C4	0.22490 (17)	0.72195 (7)	0.12872 (11)	0.0158 (2)	
C5	0.45085 (18)	0.73798 (7)	0.33979 (11)	0.0163 (2)	
C6	0.52216 (18)	0.69442 (7)	0.44890 (11)	0.0180 (2)	
H6	0.4942	0.6416	0.4469	0.022*	
C7	0.62928 (18)	0.72683 (7)	0.55577 (11)	0.0184 (2)	
H7	0.6750	0.6973	0.6276	0.022*	
C8	0.67065 (18)	0.80541 (7)	0.55709 (11)	0.0163 (2)	
C9	0.60301 (17)	0.84828 (7)	0.44820 (11)	0.0158 (2)	
C10	0.49085 (18)	0.81456 (7)	0.33800 (11)	0.0166 (2)	
H10	0.4448	0.8435	0.2655	0.020*	
C11	0.76954 (18)	0.85771 (7)	0.64502 (11)	0.0185 (2)	
H11	0.8326	0.8478	0.7286	0.022*	
C12	0.8408 (2)	0.99639 (7)	0.64371 (12)	0.0229 (3)	
H12A	0.8857	0.9907	0.7323	0.034*	
H12B	0.7370	1.0358	0.6215	0.034*	
H12C	0.9554	1.0109	0.6147	0.034*	
H3A	0.369 (2)	0.6491 (11)	0.2405 (15)	0.026 (4)*	
C13	0.3829 (3)	0.48388 (8)	0.32210 (13)	0.0322 (3)	
H13A	0.5208	0.4662	0.3597	0.048*	
H13B	0.2942	0.4400	0.2953	0.048*	
H13C	0.3367	0.5132	0.3814	0.048*	
O1	0.37868 (17)	0.53015 (5)	0.22112 (9)	0.0274 (2)	
H1	0.372 (3)	0.5011 (11)	0.1665 (19)	0.039 (5)*	

### Atomic displacement parameters (Å^2^)

**Table:**

	*U*^11^	*U*^22^	*U*^33^	*U*^12^	*U*^13^	*U*^23^
Cl1	0.0307 (2)	0.01501 (17)	0.02887 (19)	0.00241 (11)	−0.00087 (14)	0.00305 (11)
N1	0.0193 (5)	0.0211 (5)	0.0176 (5)	0.0000 (4)	0.0040 (4)	0.0018 (4)
N2	0.0163 (5)	0.0168 (5)	0.0171 (5)	0.0008 (4)	0.0029 (4)	0.0008 (4)
N3	0.0198 (5)	0.0133 (5)	0.0163 (5)	0.0013 (4)	0.0032 (4)	0.0005 (4)
N4	0.0197 (5)	0.0192 (5)	0.0134 (5)	0.0006 (4)	0.0008 (4)	0.0003 (4)
N5	0.0189 (5)	0.0195 (5)	0.0136 (5)	0.0005 (4)	0.0007 (4)	−0.0001 (4)
C1	0.0171 (6)	0.0160 (5)	0.0212 (6)	0.0000 (4)	0.0048 (5)	0.0021 (4)
C2	0.0195 (6)	0.0213 (6)	0.0178 (6)	−0.0015 (5)	0.0051 (5)	−0.0022 (4)
C3	0.0189 (6)	0.0170 (5)	0.0189 (6)	−0.0001 (4)	0.0059 (5)	−0.0009 (4)
C4	0.0142 (5)	0.0174 (5)	0.0169 (6)	−0.0011 (4)	0.0063 (5)	0.0009 (4)
C5	0.0156 (5)	0.0182 (6)	0.0155 (6)	0.0013 (4)	0.0050 (4)	0.0006 (4)
C6	0.0190 (6)	0.0161 (5)	0.0191 (6)	0.0005 (4)	0.0058 (5)	0.0033 (4)
C7	0.0191 (6)	0.0195 (6)	0.0167 (6)	0.0029 (4)	0.0052 (5)	0.0057 (4)
C8	0.0152 (5)	0.0200 (6)	0.0140 (5)	0.0022 (4)	0.0044 (4)	0.0028 (4)
C9	0.0146 (5)	0.0172 (5)	0.0156 (6)	0.0021 (4)	0.0042 (4)	0.0021 (4)
C10	0.0173 (6)	0.0181 (5)	0.0140 (6)	0.0015 (4)	0.0038 (4)	0.0021 (4)
C11	0.0182 (6)	0.0217 (6)	0.0149 (5)	0.0019 (4)	0.0035 (4)	0.0020 (4)
C12	0.0260 (7)	0.0199 (6)	0.0191 (6)	−0.0017 (5)	0.0004 (5)	−0.0024 (4)
C13	0.0489 (9)	0.0241 (7)	0.0239 (7)	0.0045 (6)	0.0109 (6)	0.0023 (5)
O1	0.0438 (6)	0.0168 (5)	0.0215 (5)	0.0015 (4)	0.0095 (4)	−0.0006 (4)

### Geometric parameters (Å, °)

**Table:**

Cl1—C1	1.7495 (13)	C6—C7	1.3641 (18)
N1—C1	1.3130 (16)	C6—H6	0.9500
N1—C2	1.3566 (16)	C7—C8	1.4125 (17)
N2—C1	1.3219 (16)	C7—H7	0.9500
N2—C4	1.3454 (15)	C8—C11	1.3933 (17)
N3—C4	1.3576 (15)	C8—C9	1.4205 (16)
N3—C5	1.4077 (15)	C9—C10	1.4124 (17)
N3—H3A	0.846 (19)	C10—H10	0.9500
N4—C9	1.3574 (16)	C11—H11	0.9500
N4—N5	1.3601 (14)	C12—H12A	0.9800
N5—C11	1.3389 (16)	C12—H12B	0.9800
N5—C12	1.4565 (16)	C12—H12C	0.9800
C2—C3	1.3638 (17)	C13—O1	1.4111 (17)
C2—H2	0.9500	C13—H13A	0.9800
C3—C4	1.4120 (16)	C13—H13B	0.9800
C3—H3	0.9500	C13—H13C	0.9800
C5—C10	1.3783 (16)	O1—H1	0.80 (2)
C5—C6	1.4312 (16)		
			
Cg1···Cg2^i^	3.6404 (9)	Cg2···Cg3^iii^	3.4566 (9)
Cg2···Cg3^ii^	3.6725 (9)		
			
C1—N1—C2	112.94 (11)	C6—C7—H7	120.8
C1—N2—C4	114.57 (10)	C8—C7—H7	120.8
C4—N3—C5	129.02 (10)	C11—C8—C7	135.37 (11)
C4—N3—H3A	115.7 (11)	C11—C8—C9	104.85 (10)
C5—N3—H3A	114.9 (11)	C7—C8—C9	119.78 (11)
C9—N4—N5	103.58 (9)	N4—C9—C10	127.40 (11)
C11—N5—N4	114.21 (10)	N4—C9—C8	111.04 (10)
C11—N5—C12	126.14 (11)	C10—C9—C8	121.56 (11)
N4—N5—C12	119.65 (10)	C5—C10—C9	117.36 (11)
N1—C1—N2	131.22 (11)	C5—C10—H10	121.3
N1—C1—Cl1	114.91 (9)	C9—C10—H10	121.3
N2—C1—Cl1	113.87 (9)	N5—C11—C8	106.32 (11)
N1—C2—C3	123.22 (11)	N5—C11—H11	126.8
N1—C2—H2	118.4	C8—C11—H11	126.8
C3—C2—H2	118.4	N5—C12—H12A	109.5
C2—C3—C4	117.26 (11)	N5—C12—H12B	109.5
C2—C3—H3	121.4	H12A—C12—H12B	109.5
C4—C3—H3	121.4	N5—C12—H12C	109.5
N2—C4—N3	119.92 (11)	H12A—C12—H12C	109.5
N2—C4—C3	120.69 (11)	H12B—C12—H12C	109.5
N3—C4—C3	119.39 (11)	O1—C13—H13A	109.5
C10—C5—N3	123.90 (11)	O1—C13—H13B	109.5
C10—C5—C6	121.15 (11)	H13A—C13—H13B	109.5
N3—C5—C6	114.93 (10)	O1—C13—H13C	109.5
C7—C6—C5	121.72 (11)	H13A—C13—H13C	109.5
C7—C6—H6	119.1	H13B—C13—H13C	109.5
C5—C6—H6	119.1	C13—O1—H1	104.9 (14)
C6—C7—C8	118.43 (11)		
			
C9—N4—N5—C11	0.26 (13)	C5—C6—C7—C8	−0.37 (18)
C9—N4—N5—C12	−179.77 (11)	C6—C7—C8—C11	179.08 (13)
C2—N1—C1—N2	1.7 (2)	C6—C7—C8—C9	−0.80 (17)
C2—N1—C1—Cl1	−179.22 (9)	N5—N4—C9—C10	178.57 (11)
C4—N2—C1—N1	0.2 (2)	N5—N4—C9—C8	−0.47 (13)
C4—N2—C1—Cl1	−178.82 (8)	C11—C8—C9—N4	0.50 (14)
C1—N1—C2—C3	−0.94 (18)	C7—C8—C9—N4	−179.58 (10)
N1—C2—C3—C4	−1.52 (18)	C11—C8—C9—C10	−178.60 (11)
C1—N2—C4—N3	177.11 (10)	C7—C8—C9—C10	1.31 (18)
C1—N2—C4—C3	−2.98 (16)	N3—C5—C10—C9	−178.85 (11)
C5—N3—C4—N2	−11.79 (18)	C6—C5—C10—C9	−0.61 (17)
C5—N3—C4—C3	168.30 (11)	N4—C9—C10—C5	−179.53 (11)
C2—C3—C4—N2	3.61 (17)	C8—C9—C10—C5	−0.58 (17)
C2—C3—C4—N3	−176.48 (11)	N4—N5—C11—C8	0.04 (14)
C4—N3—C5—C10	−16.33 (19)	C12—N5—C11—C8	−179.92 (12)
C4—N3—C5—C6	165.33 (11)	C7—C8—C11—N5	179.79 (13)
C10—C5—C6—C7	1.12 (18)	C9—C8—C11—N5	−0.32 (13)
N3—C5—C6—C7	179.51 (11)		

Symmetry codes: (i) *x*+1, −*y*+3/2, *z*+1/2; (ii) *x*−1, −*y*+3/2, *z*−1/2; (iii) *x*, −*y*+3/2, *z*−1/2.

### Hydrogen-bond geometry (Å, °)

**Table:**

*D*—H···*A*	*D*—H	H···*A*	*D*···*A*	*D*—H···*A*
O1—H1···N4^iv^	0.80 (2)	2.04 (2)	2.8394 (15)	176 (2)
N3—H3A···O1	0.846 (19)	2.110 (19)	2.9452 (14)	168.8 (16)

Symmetry codes: (iv) −*x*+1, *y*−1/2, −*z*+1/2.
